# Molecular specification of germ layers in vertebrate embryos

**DOI:** 10.1007/s00018-015-2092-y

**Published:** 2015-12-14

**Authors:** Clemens Kiecker, Thomas Bates, Esther Bell

**Affiliations:** MRC Centre for Developmental Neurobiology, King’s College London, Guy’s Campus, London, UK; Leibniz Institute on Aging, Fritz Lipmann Institute, Jena, Germany

**Keywords:** Mesendoderm, Nieuwkoop Centre, Spemann organiser, Induction, Nodal, Vg1, Activin, Wnt, FGF, TGFβ

## Abstract

In order to generate the tissues and organs of a multicellular organism, different cell types have to be generated during embryonic development. The first step in this process of cellular diversification is the formation of the three germ layers: ectoderm, endoderm and mesoderm. The ectoderm gives rise to the nervous system, epidermis and various neural crest-derived tissues, the endoderm goes on to form the gastrointestinal, respiratory and urinary systems as well as many endocrine glands, and the mesoderm will form the notochord, axial skeleton, cartilage, connective tissue, trunk muscles, kidneys and blood. Classic experiments in amphibian embryos revealed the tissue interactions involved in germ layer formation and provided the groundwork for the identification of secreted and intracellular factors involved in this process. We will begin this review by summarising the key findings of those studies. We will then evaluate them in the light of more recent genetic studies that helped clarify which of the previously identified factors are required for germ layer formation in vivo, and to what extent the mechanisms identified in amphibians are conserved across other vertebrate species. Collectively, these studies have started to reveal the gene regulatory network (GRN) underlying vertebrate germ layer specification and we will conclude our review by providing examples how our understanding of this GRN can be employed to differentiate stem cells in a targeted fashion for therapeutic purposes.

## A history of germ layers leading up to the three-signal model for mesoderm formation

**Fig. 1:**
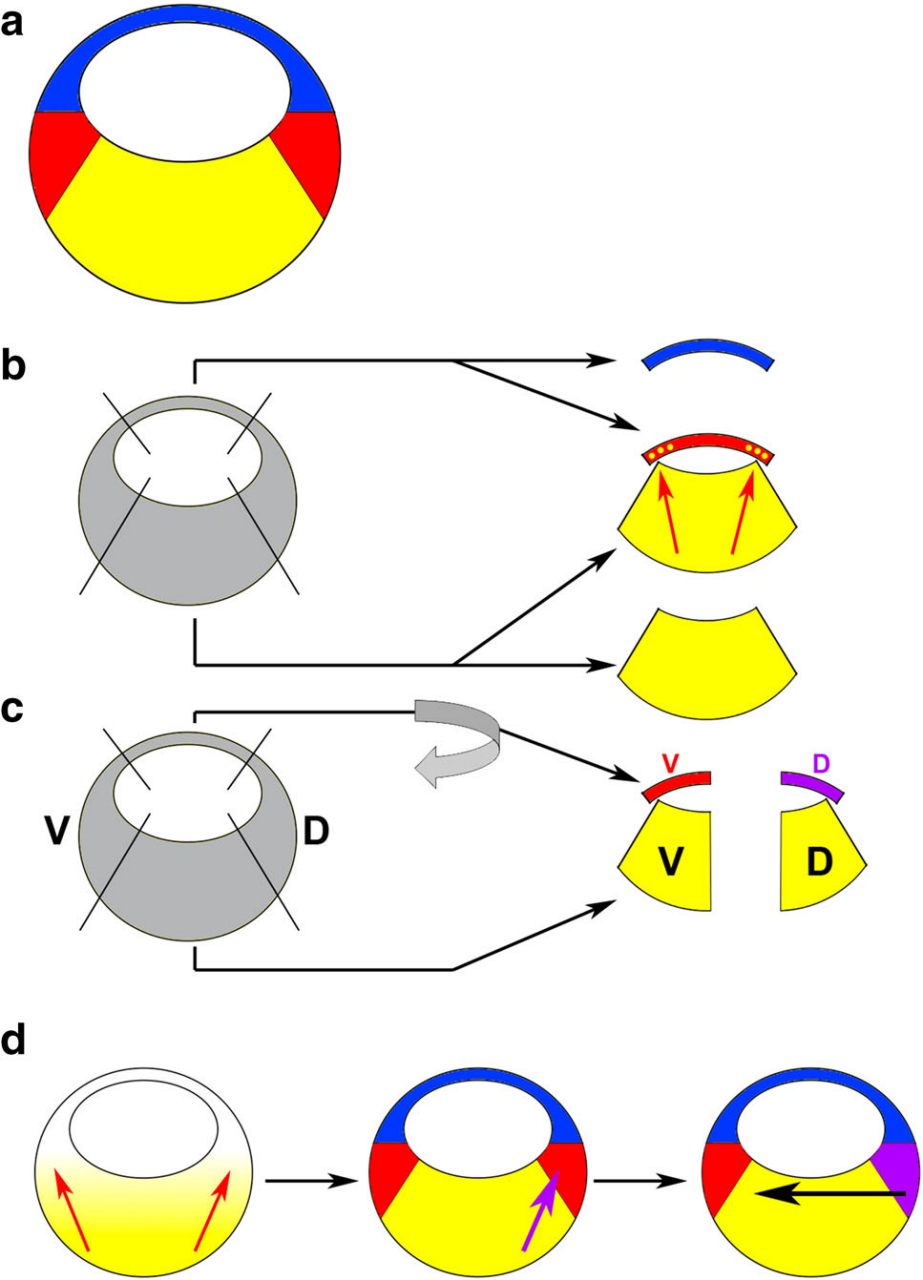
Experiments leading up to the three-signal model (3SM). **a** Schematic of an amphibian late blastula stage embryo after germ layer induction; animal points up, vegetal points down, ectoderm is shown in *blue*, mesoderm in *red*, endoderm in *yellow*. **b** When cultured in isolation, animal and vegetal explants differentiate into ectoderm and endoderm, respectively; in animal–vegetal co-cultures, mesoderm (and some endoderm) is induced in the animally derived tissue suggesting an inducing signal emanating from the vegetal tissue (*red arrows*). **c** Dorsovegetal cells induce dorsal mesoderm (*purple*) whereas ventrovegetal cells induce ventral mesoderm, even if the animal tissue is rotated by 180°. **d** The 3SM for germ layer formation: (1) a signal emanating from vegetal cells of the embryo induces the mesoderm in an equatorial ring (*red arrows*), (2) a signal from dorsovegetal cells dorsalises the mesoderm on the dorsal side (*purple arrow*) and (3) signals from the dorsal mesoderm (Spemann’s organiser) pattern the embryo along its DV axis (*black arrow*). *D*, dorsal; *V*, ventral

In the second half of the eighteenth century Caspar Friedrich Wolff noted that the cells of an embryo are organised in layers, and this observation formed the foundation of the concept that embryos consist of germ layers, developed in the nineteenth century by Heinz Christian Pander. The end of the nineteenth century was marked by the advent of experimental embryology and, based on their famous grafting experiments in amphibians, Hans Spemann and others established the concepts of embryonic induction and competence. Somewhat surprisingly, germ layer formation attracted relatively little interest at that time and the work of the embryologists focused on the formation of more definitive tissues such as the brain, limbs and eyes. However, in his classical fate mapping studies Vogt already mapped the origin of the mesoderm to the involuting marginal zone (“Randzone”) of the gastrula of different amphibian species (Fig. 1a) [1]. This led researchers to realise that the germ layers become established during the process of gastrulation.

In the first half of the twentieth century, the search for embryonic inducers led to occasional cases of mesoderm induction [2]. The inducers in these studies were typically heterologous (extracted from adult tissues, often of other species) and thus their biological relevance remained questionable. It was only in the 1960s and 1970s that Pieter Nieuwkoop and his colleagues published a series of seminal studies that identified mesoderm specification as an inductive process between the vegetal cell mass (endoderm) and the animal cap (ectoderm) of a blastula stage salamander embryo. Specifically, Nieuwkoop found that vegetal cells form endoderm and animal cap explants form ectoderm when they were cultured in isolation, but that mesoderm was induced when both types of tissues were co-cultured as aggregates [3]. Nieuwkoop could also show that not only mesoderm, but also pharyngeal endoderm were induced in the co-cultures, and that the induced mesendoderm (cells that can differentiate into both endoderm and mesoderm) was entirely derived from the animal cap component of the aggregates, suggesting that an inducing signal emanates from vegetal cells (Fig. 1b) [3]. Therefore, it was clear that mesoderm and endoderm formation require inductive signals.

In these experiments, Nieuwkoop also showed that there is a dorsoventral (DV) bias within the vegetal cell mass such that dorsovegetal blastomeres induce dorsal mesoderm whereas ventrovegetal blastomeres induce ventral mesoderm (Fig. 1c) [4]. The dorsal marginal zone (DMZ) is where the involution movements of gastrulation begin, and Spemann and his colleagues had demonstrated previously that the transplantation of tissue from this region at the onset of gastrulation to the ventral side of a host embryo resulted in the formation of a second body axis. The ectopically induced twins contained only few cells that came from the grafted DMZs and most of their tissue was derived from the host embryos, indicating that the early gastrula DMZ can organise the formation of a fully patterned embryo in surrounding tissue [5]. These experiments coined the term ‘Spemann organiser’ for the early gastrula DMZ and won Spemann the Nobel Prize in Medicine in 1935. Nieuwkoop’s studies suggested that the dorsal mesoderm including Spemann’s organiser is induced by dorsovegetal endodermal cells, and this “organiser of the organiser” is often referred to as the ‘Nieuwkoop Centre’.

These and other experiments led to the ‘three-signal model’ (3SM) for mesoderm formation (Fig. 1d): (1) vegetal cells secrete a mesendoderm-inducing factor that converts the marginal zone into a ring of mesoderm, (2) the vegetal cell mass is subdivided into dorsal and ventral parts that induce dorsal and ventral mesoderm, respectively, and (3) the most dorsal mesoderm (Spemann’s organiser) secretes signals that establish DV polarity by promoting dorsal identity [6–8]. The first two steps are thought to occur early in development, at the onset of zygotic transcription, whereas Spemann’s organiser operates at a later stage, during gastrulation. The Nieuwkoop Centre contributes to the second signal in this model—it acts upstream of Spemann’s organiser. In this review we will focus on the initial induction of the germ layers (corresponding to the first two steps of the 3SM). The inductive effects of the Spemann organiser and the secondary patterning of the germ layers (including the important role of the organiser in ectodermal regionalisation) have been reviewed elsewhere in several excellent reviews [9–13].

It is debatable to what extent the first two steps in the 3SM can be uncoupled. Nieuwkoop himself initially favoured a model whereby a single mesendoderm inducer is released in a graded fashion—high levels dorsally and low levels ventrally. However, experiments using irradiation of amphibian embryos with ultraviolet (UV) light pointed towards two independent signals mediating mesendoderm induction and dorsal specification: mesendoderm induction was generally unaffected in such embryos, but they displayed dorsoanterior defects suggesting that only the second signal of the 3SM was affected [14–16].

## Secreted factors as candidate mesendoderm inducers

Nieuwkoop’s finding that mesoderm formation depends on an inductive event suggests that it is mediated by (an) extracellular factor(s) that is secreted from vegetal cells at the blastula stage. Since the late 1980s, several candidates for the first and second signal have been identified and characterised.

### Fibroblast growth factors

The first purified protein shown to induce mesoderm in amphibian animal cap tissue was basic fibroblast growth factor (bFGF) [17]. These experiments were performed using embryos of the African claw-toed frog *Xenopus laevis*. Shortly thereafter the first *Xenopus fgf* gene, *bfgf*, was cloned and the protein shown to be present at biologically active levels in the oocyte [18]. Since then multiple studies demonstrated mesoderm-inducing activity for FGFs and for many of its downstream signal transducers in frog [19–22], fish [23], chick [24] and mouse embryos [25]. Blocking FGF function in these embryos leads to mesodermal defects of varying degrees [19, 20, 22, 24, 26, 27].

Despite this overwhelming evidence for an important role in mesoderm induction, FGFs alone are unlikely to represent Nieuwkoop’s inducer of mesoderm and endoderm. First, there is no evidence for the induction of endoderm by FGFs; second, *fgf* mRNA is expressed in the marginal zone at early gastrula stages, in the prospective mesoderm of the frog embryo, rather than in vegetal cells [28, 29]; and third, mesoderm is affected, but never completely eliminated in embryos with loss of FGF signalling function, suggesting that other factors at least partially compensate for the lack of FGFs. In zebrafish, FGFs were found to regulate DV patterning of the mesoderm rather than its induction, i.e., the third signal of the 3SM rather than the second [30, 31]. A role for FGFs in DV patterning has recently also been suggested in *Xenopus* [32].

Several studies in frog and fish embryos proposed that, rather than being instructive inducers of mesodermal fate, FGFs function as competence factors that are required for the cellular response to another group of mesendoderm inducers, the transforming growth factor βs (TGFβs) [33–37]. It has also been proposed that FGFs act secondarily to set apart the mesoderm from the TGFβ-induced mesendoderm [38]. Taken together, it is clear that FGF signalling plays an important role in mesoderm formation, but it is not sufficient for germ layer formation on its own.

### Activin

Around the time when FGFs were discovered as potential mesoderm inducers, TGFβs were also found to induce mesoderm [33]. The first TGFβ factor coming into play was Activin A [39–41]. Activin, in addition to being able to induce a secondary axis [42], can induce a range of different DV mesodermal cell fates in a dose-dependent manner, consistent with the graded mesoderm inducer initially proposed by Nieuwkoop (see above) [42, 43]. Unlike FGFs, Activin also induces endoderm [39, 44, 45]. Activin was also shown to function as a mesendoderm inducer in chick and zebrafish [46, 47]. However, doubts about Activin’s candidacy as the primary mesendoderm inducer were raised (1) by the failure of the Activin inhibitor Follistatin to interfere with mesoderm induction in frog embryos [48] (but see [49]) and (2) by the relatively mild phenotype of mouse embryos with disrupted *Activin* genes which suggested that this factor is not endogenously required for mesendoderm formation [50]. Nonetheless reducing levels of Activin using morpholino antisense nucleotides was shown to affect mesoderm formation to at least some extent in the frog embryo more recently [51, 52].

Attempts to interfere with Activin signalling downstream of the ligand—for example by inhibiting Activin receptor function—often resulted in much stronger defects of mesendoderm formation compared to experimental removal of the ligand itself [53]. The most likely reason for this effect is that other TGFβ ligands, which may also be involved in mesendoderm formation, signal via the same receptor pathway.

### Vg1

One of these ligands is Vg1, which was discovered as a vegetally localised mRNA in the *Xenopus* embryo. In fact, this factor initially attracted interest as a model for mRNA localisation in oocytes [54]. Like other TGFβs, Vg1 is produced as a precursor peptide that needs to be cleaved and dimerise to become active. Somewhat perplexingly, although the Vg1 precursor was found to be abundant in early embryos, its mature form could not be detected. Furthermore injection of wild-type *Vg1* mRNA failed to produce the axial duplications expected for a bona fide mesendoderm inducer like Activin, and only synthetic constructs in which the prepro-region (the N-terminal domain of the unprocessed polypeptide) of bone morphogenetic proteins 2 or 4 (BMP2/4) was fused to the core domain of mature Vg1 resulted in Activin-like effects [55, 56]. These results suggested that the conversion of Vg1 into its active form is highly inefficient, and that only tiny amounts of the active protein are present in the developing embryo. This could either mean that active Vg1 is so potent that its levels need to be kept extremely low, or that Vg1 is not the endogenous mesendoderm inducer.

Recently the Heasman lab was able to resolve the conundrum of the seemingly inactive Vg1 by demonstrating—using antisense depletion in *Xenopus*—that Vg1 is indeed essential for (dorsal) mesoderm formation. They were also able to show that the protein encoded by a second *Vg1* gene with a proline → serine substitution in its pro-domain was much more efficiently processed than the original Vg1 and is therefore biologically active [57]. This study explains the effects obtained previously with a dominant-negative variant of Vg1: apparently this dominant-negative simultaneously antagonised both versions of Vg1, resulting in severe defects in mesoderm and endoderm formation [58].

Vg1 in chick and its zebrafish orthologue Dvr1 are also expressed early in development and possess mesoderm-inducing activity [59–61]. Yet morpholino-mediated knockdown of Dvr1 in zebrafish embryos affected asymmetric development along the left–right axis, but not mesoderm formation as such [62]. In the mouse embryo, the Vg1 orthologue Gdf1 appears to synergise with its close relative Gdf3 since *Gdf1*^−/−^*;Gdf3*^−/−^ double mutants are more severely affected than either single mutant, with distinctive defects in mesoderm and endoderm formation [63].

A close relative of Vg1, Derrière (orthologue of mammalian *Gdf3*), was found to be expressed in the future endoderm and mesoderm at late blastula stages of *Xenopus* development. Gain- and loss-of-function experiments with Derrière pointed towards a role in the induction of the posterior mesendoderm [64]. Thus, both Activins and Vg1/Dvr1/Gdf1/3 are candidate TGFβs involved in mesendoderm formation; however, their relevance may vary between different species and loss-of-function approaches have only partially substantiated a role for these factors as endogenous mesendoderm inducers.

### Nodals

Genetic loss-of-function studies in mouse and zebrafish introduced the Nodal subfamily of TGFβ factors as mesendoderm inducers *par excellence*. These studies revealed that Nodal in the mouse and the Nodal-like factors Cyclops and Squint in zebrafish are strictly required for mesoderm and endoderm formation [65–67]; reviewed in [68, 69]. Similarly inactivation of *ActRIB* (encoding a Nodal receptor), of genes encoding Nodal co-receptors of the EGF-CFC (epidermal growth factor-Cripto/FRL1/Cryptic) family, and of the intracellular TGFβ signal transducers *Smad2,Smad3* and *Smad4* lead to severe defects in mesendoderm induction [22, 70–74].

Blocking Nodal signalling in *Xenopus* proved somewhat more difficult due to the large number of Nodal ligands expressed at late blastula stage [75–78]. However, simultaneous morpholino antisense knockdown of *Xenopus nodal*-*related* (*nr*) *5* and *6* resulted in mesendoderm specification defects suggesting that these two may be the major players in this process, consistent with them being the earliest expressed *nrs* [52, 79].

Nodal signals are not only essential for mesendoderm induction; they also function as potent mesoderm and endoderm inducers in gain-of-function experiments [21, 75, 77]. So, are Nodals the primary mesendoderm inducers in vertebrate embryos? A subset of *Nodal*^−/−^ mouse embryos express genetic markers of the definitive posterior mesoderm and, similarly, ventroposterior mesoderm is found in *squint;cyclops* double mutant zebrafish embryos [66, 67]. This suggests that, even in complete absence of Nodal signalling, there is some residual mesendoderm-inducing activity present in vertebrate embryos. No mesoderm was found in *Smad2*^−/−^*;Smad3*^−/−^ double mutant mice indicating that this residual activity is likely mediated by other Nodal-type molecules such as Gdf1 and Gdf3, rather than the above mentioned FGFs [74].

### Bone morphogenetic proteins

BMPs are also members of the TGFβ family that have anecdotally been implicated in mesoderm induction. However, compared to the Nodal subfamily, BMPs are poor mesoderm inducers [80]. BMPs signal via a different branch of the TGFβ pathway that involves Smad1 and Smad5, rather than Smad2 and Smad3, and it is possible that their weak mesoderm-inducing activity is due to aberrant activation of the Smad2/3 branch of the pathway following non-physiological overexpression. It is now broadly accepted that a major role of BMPs is to regulate DV patterning of the mesoderm, i.e., the third step of the 3SM.

Thus, the TGFβ signalling pathway is crucial in mesendoderm induction. The concentration effect of the different ligands (Nodal/Activin/Vg1) results in the different responses (mesoderm/endoderm). However, the relative importance of each ligand may differ between different vertebrate species, and it remains to be established whether they simply function in an additive fashion, or whether they exert qualitatively different effects. FGFs seem to function by establishing the competence for mesoderm induction by TGFβ signals, rather than by instructing mesodermal fates themselves.

### Maternal Wnt/β-catenin and the establishment of dorsal identity

The first indication that signalling factors of the Wnt family could be involved in inducing the ‘Nieuwkoop Centre’ came from early overexpression studies in *Xenopus* where injection of *Wnt* mRNAs into ventro-vegetal blastomeres of the early embryo frequently resulted in complete axial duplications highly reminiscent of the Spemann organiser grafting experiment [81, 82]. In fact, this ‘axis induction essay’ played a key role in establishing the canonical Wnt signalling pathway in vertebrates in the 1990s [83–87].

**Fig. 2:**
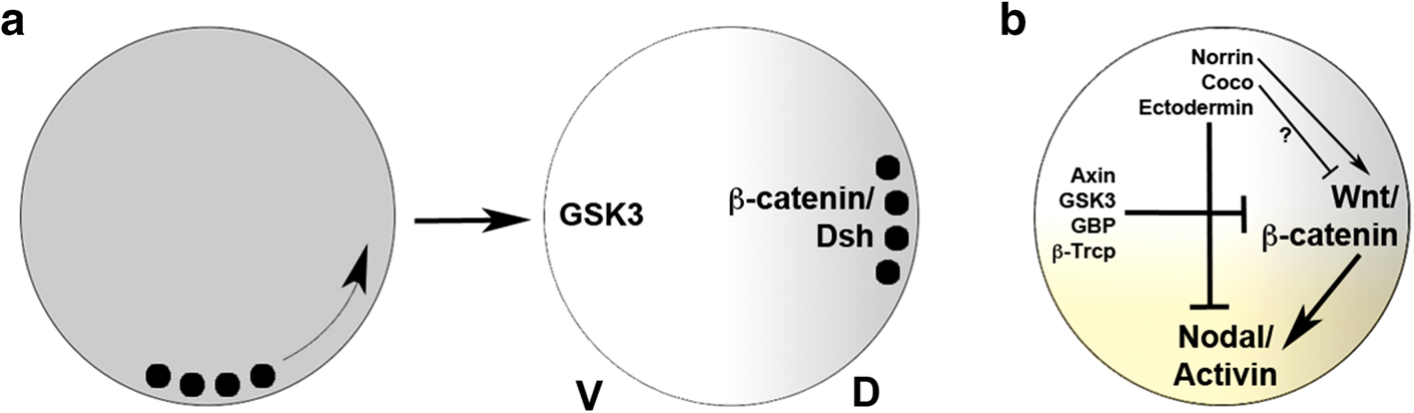
The establishment of orthogonal axes in the amphibian blastula embryo. **a** Cortical rotation transports vegetal determinants (*black dots*) to the future dorsal side of the embryo leading to enrichment of β-catenin and Dishevelled (Dsh) and downregulation of GSK3 dorsally. **b** Wnt/β-catenin signalling (*grey* gradient) specifies the dorsal side and vegetal Nodal/Activin signalling induces endoderm and mesoderm (*yellow* gradient). Wnt signalling is antagonised by the Wnt destruction complex involving Axin and GSK3 whereas Coco, Ectodermin and Norrin antagonise Nodal/Activin signalling in the animal hemisphere. Norrin also promotes, whereas Coco may inhibit, Wnt signalling

As mentioned above, Nieuwkoop Centre induction is sensitive to UV light such that irradiated embryos develop lacking dorsal characteristics. Fertilisation of the amphibian egg triggers a rotation of the egg cortex relative to its cytoplasm [15], and UV irradiation was shown to block this cortical rotation. The cellular target affected by UV light is a dense array of microtubules in the vegetal cortex of the egg [88], suggesting that maternal determinants that are initially found at the vegetal pole of the egg become actively translocated to the dorsal side by cortical rotation. Injection of *Wnt* mRNA was shown to rescue the effects of UV light; however, the first true link between the Wnt signalling pathway and axis determination came from the observation that β-catenin, an intracellular transducer of Wnt signals, becomes enriched in the dorsal half of the embryo and that this enrichment may involve active transport along microtubules and/or selective protein stabilisation [89, 90]. Not only β-catenin, but also Dishevelled—an adaptor protein that mediates Wnt signalling downstream of its receptor and upstream of β-catenin—and glycogen synthase kinase 3 (GSK3) binding protein (GBP) are transported to the dorsal side [91, 92]. Simultaneously, GSK3, an intracellular antagonist of the Wnt pathway, becomes down-regulated dorsally (Fig. 2a) [93].

Whether the maternal dorsalising pathway is actually activated by a Wnt ligand has been controversial. Of the three Wnt ligands that are maternally expressed in *Xenopus*, Wnt8B and Wnt5A do not seem to be localised dorsally, and Wnt8B is only expressed at extremely low levels [83, 94]. Furthermore, Wnt5A and Wnt11 belong to a different class of Wnt ligands that are less efficient in activating the canonical Wnt signalling pathway and tend to stimulate an alternative pathway which affects morphogenesis and may even antagonise the canonical Wnt pathway [95, 96]. Genetic mutants of *Wnt5A* and *Wnt11* in zebrafish and mouse also support a role for these factors in regulating morphogenesis rather than early DV patterning [97–99]. Moreover, inhibitors that block Wnt signalling extracellularly by ligand sequestration or by antagonising the Wnt receptor complex frequently failed to affect DV axis formation [96, 100]. Taken together, these studies initially suggested that the maternal dorsalising signal may not involve a Wnt ligand, but rather depend on pathway activation at the intracellular level. However, more recently the Heasman lab found that maternal *Wnt11* mRNA is indeed required and sufficient to activate the dorsalising pathway in *Xenopus* [101]. Wnt11 is the only Wnt ligand that shows a dorsal enrichment at cleavage stages [102], and its interaction with the maternally expressed receptor Frizzled-7 results in canonical pathway activation [103]. The axis-inducing activity of maternal Wnt11 depends on heparan sulphate proteoglycans and on FRL1, a member of the EGF-CFC co-receptor family that is also essential for Nodal signalling [101]. Since EGF-CFCs were initially identified as atypical FGF receptor ligands [104], it is tempting to speculate that these factors somehow integrate multiple maternal signals that are involved in germ layer specification.

Follow-up studies revealed that both Wnt5A and Wnt11 synergise in this process and that they function as homodimers in mediating the early dorsalising signal [105, 106]. Furthermore, Lipoprotein receptor-related protein 6 (LRP6) is required for this signal, and the LRP6 antagonist Dickkopf 1 (Dkk1) is present as a maternal mRNA required to prevent excessive Wnt signalling at this stage [105, 107]. Thus, the maternal dorsalising signal has many characteristics of the canonical Wnt/β-catenin signalling pathway, but also several unusual features (Wnt5A/Wnt11 activating the canonical pathway; Wnts as homodimers; the involvement of FRL1).

There is now evidence that the maternal dorsalising pathway is activated more broadly than just in dorsovegetal blastomeres: studies in *Xenopus* indicated that the maternal Wnt/β-catenin-dependent pathway induces neural fate in the dorsal ectoderm [108, 109]. This happens via two parallel routes: transcriptional repression of *Bmp2* [108] and induction of the extracellular BMP inhibitors Chordin, Noggin and Cerberus [109, 110]. BMP signalling promotes ventral identity in all germ layers and the interplay between BMP inhibitors dorsally and BMPs ventrally is thought to generate a BMP activity gradient that regulates DV patterning of the embryo during gastrulation—the third step of the 3SM [9, 11–13].

Thus, maternal Wnt/β-catenin signalling may be active throughout the dorsal hemisphere of the *Xenopus* blastula, and mesendoderm induction and the establishment of dorsal identity (steps 1 + 2 of the 3SM) occur—at least in part—independently. In this scenario maternal Wnt/β-catenin signalling on its own does not strictly qualify for Nieuwkoop’s second signal, as it is not limited to the vegetal blastomeres of the embryo. It rather seems to be the case that two overlapping signalling systems—Nodal/Activin and Wnt/β-catenin—specify the animal–vegetal and DV axes of the embryo (Fig. 2b). These two signalling pathways are linked, as early Wnt signalling induces the *Nodal* genes *Xenopus nodal*-*related 3, 5* and *6* (*nr3*/*5*/*6*) [72, 76–78, 111, 112]. Nr3 is an atypical Nodal ligand that antagonises TGFβ signalling [113], but nr5 and nr6 initiate a cascade of *Nodal* expression that is central to mesendoderm specification [79, 112]. A Wnt responsive element has been identified in the *nr1* promoter, further supporting the idea of an interaction of the two pathways [114].

A study demonstrating that maternal β-catenin is not only active dorsally but also required all around the equatorial region of the embryo for mesoderm induction is somewhat at odds with the discussed role of this signalling pathway in dorsal specification [115]. Subtle differences in the timing of the activity of this signal may explain this finding.

## Dose-dependency in mesendoderm induction

Nieuwkoop’s observation that a mesendoderm inducer is released from vegetal blastomeres suggests that this inducer could function dose-dependently along the vegetal-animal axis, resulting in endoderm formation at highest levels, mesoderm formation at lower levels and ectoderm formation in the absence of the inducer (Fig. 2b). This model is supported by studies in zebrafish where graded Nodal signalling patterns the germ ring—the marginal zone of the fish embryo where all mesendodermal progenitors are located—along the vegetal-animal axis [116–118]. Evidence for a vegetal–animal gradient of Nodal/Activin signalling in amphibians is sparse and mostly indirect, based on experiments using factors that inhibit different TGFβ-type factors with different efficiencies [119]. The large number of different TGFβ ligands expressed in pre-gastrula stage *Xenopus* embryos makes the interpretation of such experiments particularly difficult.

There is, however, fairly good evidence that Nodal/Activin signalling in *Xenopus* is biased along the DV axis with higher levels specifying dorsal, and lower levels specifying ventral fates. In gain-of-function experiments, Activin (and later Nodal and Vg1) was shown to induce different mesodermal cell fates along the DV axis in a dose-dependent manner [43, 75, 120]. The *Xenopus* Nodal genes *nr1,nr2, nr4, nr5* and *nr6* are expressed more strongly dorsally than ventrally at late blastula stage [77, 78, 111]; however, the evidence for a requirement for graded Nodal/Activin signalling in DV patterning is somewhat less conclusive, presumably due to the large number of TGFβ ligands in frogs. Injections of different doses of the synthetic Nodal inhibitor Cerberus-short (Cer-S) resulted in dorsoventrally biased effects with ventral mesoderm being affected at low doses and dorsal mesoderm at higher doses [111]. Similar dose–response experiments in zebrafish using Activin and the Nodal inhibitor Antivin, respectively, suggested that a gradient of these signals establish the anteroposterior (AP) axis of the fish [121]. However, these experiments are difficult to interpret, since the effects of TGFβ signalling on germ layer formation occur early, before gastrulation, whereas the AP axis is established during gastrulation involving complex interactions between the previously formed germ layers.

## The timing of mesendoderm induction

Because germ layer formation takes place so early in development, one of the key questions is to which extent it is maternally controlled. Activin and Vg1/Dvr1 are present as maternal factors in frog and fish embryos [46, 122]. Furthermore, the symmetry-breaking event that generates DV polarity is maternal, as it is triggered by sperm entry and involves the translocation of a maternal factor (possibly Wnt11) to the future dorsal side of the egg (see above). Yet, Nodals, the major class of mesendoderm-inducing factors conserved across vertebrates, are expressed zygotically in *Xenopus* embryos [78, 111]. However, zygotic expression of *nr5* and *nr6* begins at 256-cell stage—much earlier than that of many other genes—and is controlled by maternal β-catenin [72]. This early nr5/6 activity induces *nr1* and *nr2*, resulting in a cascade of Nodal expression that is at least transiently stronger on the dorsal side of the embryo [78].

Developmental signalling factors often perform different functions at different developmental stages. Thus, it is conceivable that Nodal/Activin-like factors dynamically regulate different aspects of mesendoderm induction and/or patterning in the few hours during which this process takes place in amphibians and fish. A true appreciation of the signalling dynamics of the mesendoderm-inducing factors can only come from a detailed spatiotemporal analysis of their expression or a readout of their signalling pathways.

## Activation of signalling pathways in vivo

As mentioned above, Nodal/Activin-type signals are transduced via Smad2 and Smad3. Activation of these Smads is mediated via phosphorylation; thus, immunohistochemical detection of phosphorylated Smad2/3 (p-Smad2/3) provides a way of detecting Nodal pathway activation in situ. A DV gradient of p-Smad2 can be detected in the late blastula embryo of *Xenopus*, consistent with the stronger induction of *nrs* on the dorsal side of the embryo by β-catenin [123, 124]. However, this graded signal appears to be highly transient: Fauré et al. (2000) found no significant p-Smad2 before the onset of zygotic transcription [123] whereas Schohl and Fagotto (2002) observed weak activation in a supra-equatorial ring—some distance away from the maternal TGFβ ligands Activin and Vg1 [124]. During gastrulation the DV bias in Smad2 activation seems to be lost, but overall levels remain high in the endoderm [124].

In a recent study, two different approaches were used to monitor Smad2 activation in zebrafish embryos: Smad2 nuclear localisation and Smad2–Smad4 complex formation [125]. These experiments confirm the previously postulated vegetal–animal gradient of Nodal activity in zebrafish [116–118], but they also revealed that Smad2 activation is biased along the DV axis of the fish embryo, similar to what has been observed in frogs. Thus, the role of Nodal/Activin-Smad2 signalling appears to be fairly conserved across anamniote vertebrates, contributing to both vegetal–animal and DV patterning of the mesendoderm.

Recently, this approach of monitoring Smad2 activation has been used in the zebrafish embryo to analyse the dynamics of Nodal target gene induction, revealing that not only the dose of Nodal, but also the timing and magnitude of the induction of its target genes shape the response of a tissue to this morphogen [126].

Similar to p-Smad2 serving as an indicator of Nodal/Activin signalling, phosphorylation of mitogen-activated protein kinase (p-MAPK) can be used to visualise activation of tyrosine kinase receptors, including those that activate the branch of FGF signalling that is involved in mesoderm induction. Experimental manipulation of the FGF pathway in *Xenopus* embryos alters p-MAPK distribution, indicating that p-MAPK distribution represents FGF pathway activation in vivo [127]. Endogenously activated MAPK is detected in the prospective mesoderm in the marginal zone at late blastula and gastrula stages, consistent with both its proposed role as a mesodermal competence factor and the endogenous expression of FGFs at this stage of embryonic development. Interestingly, p-MAPK shows a DV bias with higher levels of expression dorsally [124, 127, 128]. A role for FGF signalling in mesodermal DV patterning has not been proposed in the frog embryo; however, it has been suggested that FGF8 induces a DV axis in zebrafish [31]. Lower levels of p-MAPK were also found in the prospective endodermal cells at the vegetal pole of the embryo, although FGFs do not appear to play an obvious role in endoderm formation [124, 129].

The nuclear localisation of β-catenin is indicative of canonical Wnt pathway activation. Consistent with its role as the early dorsalising signal, nuclear accumulation of β-catenin is found on the dorsal side of frog and fish embryos from early blastula stages onwards [89, 124].

Thus, the activation patterns of the signalling pathways involved in mesendoderm induction are consistent with what was postulated based on gain- and loss-of-function analyses: a maternal Wnt/β-catenin signal determines the future dorsal side of the embryo whereas zygotic Nodal/Activin signalling is crucial for mesendoderm formation, and this signal also imparts DV specification as it is stronger on the dorsal side of the embryo. Simultaneously, FGF is important for the induction of the mesoderm, and this signal may also be dorsoventrally biased. FGF, Nodal/Activin and Wnt signalling are linked at multiple levels: as mentioned above, β-catenin induces the expression of Nodal ligands on the dorsal side of the embryo [72, 78], and a recent study revealed that FGF-MAPK signalling leads to N-terminal phosphorylation of the tumour suppressor protein p53 which subsequently interacts with Smads, thereby promoting Nodal/Activin signalling [130].

## Specification of the ectoderm: Nodal, FGF and Wnt inhibitors restrict mesendoderm-inducing signals

Cell fate decisions in developing embryos are typically regulated by a finely tuned interplay between inducing signals and their inhibitors. Thus, it is not surprising that inhibitors also control the signals that underlie the first step of cellular specification in embryos.

### TGFβ antagonists

The *Cerberus*/*Dan* gene family encodes multifunctional inhibitors of BMP, Nodal/Activin and Wnt signalling. One member of this family, *Coco*, is expressed maternally in *Xenopus* embryos with higher levels of expression throughout the animal hemisphere [131]. Antisense-mediated knockdown of maternal *Coco* mRNA resulted in an expansion of endoderm at the expense of mesoderm, and this effect could be rescued to differing degrees by eliminating either Activin or nr5 and nr6 [52]. These results not only suggest that the role of maternal Coco is to limit endoderm induction by high levels of Nodal/Activin signalling, they also provide evidence that Activin, nr5 and nr6 function redundantly in endoderm induction. Interestingly, the expansion of endoderm is much more noticeable on the dorsal side of the embryo, in line with the idea of a DV gradient of Nodal activity (see above).

It remains to be established whether Coco also antagonises the dorsalising Wnt5A/11 signal that establishes the ‘Nieuwkoop Centre’; however, the extent of Spemann’s organiser is reduced in Coco knockdown embryos due to the overall reduction in mesoderm formation, complicating the assessment of ‘dorsalisation’ in such embryos. The reduction of organiser activity explains the lack of anterior specification in Coco-deficient embryos—which lack heads—at later stages [52]. It would be interesting to analyse whether embryos show increased nuclear enrichment of β-catenin following Coco knockdown.

A recent study revealed that the *Xenopus* orthologue of the human disease gene *Norrin* is maternally expressed in animal blastomeres and that its overexpression results in an expansion of the neural plate (= dorsal ectoderm) [132]. It had previously been shown that Norrin activates canonical Wnt signalling by interacting with the Wnt receptor Frizzled4; thus, a dorsalised phenotype upon Norrin overexpression was not too surprising [133]. However, the authors discovered an additional function of Norrin in inhibiting Nodal/Activin and BMP signalling through direct binding and sequestration of these ligands. Thus, Norrin functions in a similar manner to Coco with respect to TGFβ signalling, but in an opposite manner with respect to Wnt signalling [132].

Another factor that is expressed maternally throughout the animal hemisphere of the *Xenopus* embryo is Ectodermin, a RING-type ubiquitin ligase that targets Smad4 for proteasomal degradation [134]. Weak expression, with a dorsal bias, is seen at gastrula stages; however, Ectodermin expression is lost after gastrulation. Smad4 is an essential co-factor for both Smad2/3 and Smad1/5; thus, Ectodermin antagonises both Nodal/Activin and BMP signalling. Antisense-mediated knockdown of maternal Ectodermin reveals essential functions in both pathways, as both endoderm and non-neural ectoderm expand at the cost of mesoderm and neuroectoderm, respectively. Taken together both extracellular and intracellular inhibitors of TGFβ signalling are required to antagonise mesendoderm induction and thereby protect the prospective ectoderm.

### FGF antagonists

No specific secreted FGF antagonists have been identified to date; however, FGF signalling is limited through a negative feedback loop that involves auto-induction of intracellular FGF inhibitors of the Sprouty and Spred families. In *Xenopus* these two families appear to differentially regulate different branches of the FGF signalling pathway: gain- and loss-of-function experiments targeting Sprouty1 and Sprouty2 revealed their role in modulating the FGF-Ca^2+^-PKCδ signalling pathway and gastrulation movements whereas a comparable set of experiments targeting Spred1 and Spred2 indicated that they antagonise MAPK activation and mesoderm specification [135]. *Sprouty* and *Spred* genes are zygotically induced by FGF signalling; thus, their role is to limit the signal after its onset, rather than excluding FGF signalling from a pre-specified domain.

### Wnt antagonists

The maternal Wnt/β-catenin signal that defines the dorsal half of the embryo is restricted by both extracellular and intracellular antagonists. The Heasman laboratory found that Dickkopf1 (Dkk1), an antagonist of the Wnt receptor complex, is present as a maternal mRNA in *Xenopus* oocytes and that its depletion using antisense oligonucleotides results in profound patterning and gastrulation defects [105]. However, this study does not explain why injection of exogenous Dkk1 mRNA into early blastula stage embryos leads to dorsoanteriorised embryos, consistent with an inhibition of Wnt signalling after the onset of zygotic transcription rather than ventralisation [136].

The maternal Wnt/β-catenin signal is also antagonised by a number of intracellular pathway inhibitors. GSK3 is part of a protein complex that targets β-catenin for destruction by the proteasome and gain- and loss-of-function experiments demonstrated an essential role in regulating primary DV patterning of the frog embryo [84, 86]. The finding that lithium ions (Li^+^) inhibit GSK3 for the first time provided an explanation as to why Li^+^-treated *Xenopus* embryos become hyperdorsalised [137]. Subsequently other components of this β-catenin destruction complex were also found to affect embryonic axis formation: the adaptor proteins Axin and Axil, the GSK3 inhibitor GBP (GSK3 binding protein) and the ubiquitin ligase β-Trcp all inhibit Wnt/β-catenin and antagonise Nieuwkoop Centre formation [87, 138–144]. Moreover, GSK3 protein levels are specifically downregulated on the dorsal side of the embryo following cortical rotation [93]. This finding suggests that Dsh and/or GBP, or an as yet unknown GSK3 antagonist, is moved towards the dorsal side of the embryo, most likely via the microtubule network mentioned above.

Taken together, all three major signalling pathways that govern the early steps of germ layer specification are antagonised at different levels. FGFs are controlled via a negative feedback loop involving Spreds; Wnt/β-catenin signalling is negatively regulated by maternal Dkk1, Norrin and by various components of the β-catenin destruction complex; and Nodal/Activin signalling is restricted to the vegetal hemisphere by maternal factors in the animal hemisphere—Coco, Norrin and Ectodermin (Fig. 2b).

## Transcription factors in germ layer specification

### VegT

In the fruit fly *Drosophila*, a classical model organism for developmental geneticists, embryonic axis formation is regulated by maternal mRNAs that are differentially localised in the oocyte. In 1996, maternal transcripts of the *Xenopus* T-domain transcription factor VegT (also known as Antipodean, Brat, Xombi or tbx6) were found to localise to the oocyte’s vegetal cortex [21, 145–147]. Ectopic expression of VegT results in induction of mesodermal [21, 145, 146] as well as endodermal markers [147] and even ectopic bottle cells—dorsovegetal cells that appear at the onset of gastrulation and mark the initial site of tissue internalisation [148]. Importantly, antisense depletion of maternal *VegT* mRNA resulted in embryos that lacked endoderm, showed a reduction and vegetal shift of the mesoderm and displayed an expansion of the ectoderm into the equatorial region, suggesting that maternal VegT is a key determinant in germ layer specification [107, 149]. In support of this, VegT-depleted vegetal poles could not induce mesoderm in co-cultured blastula stage animal cap tissue [149].

**Fig. 3:**
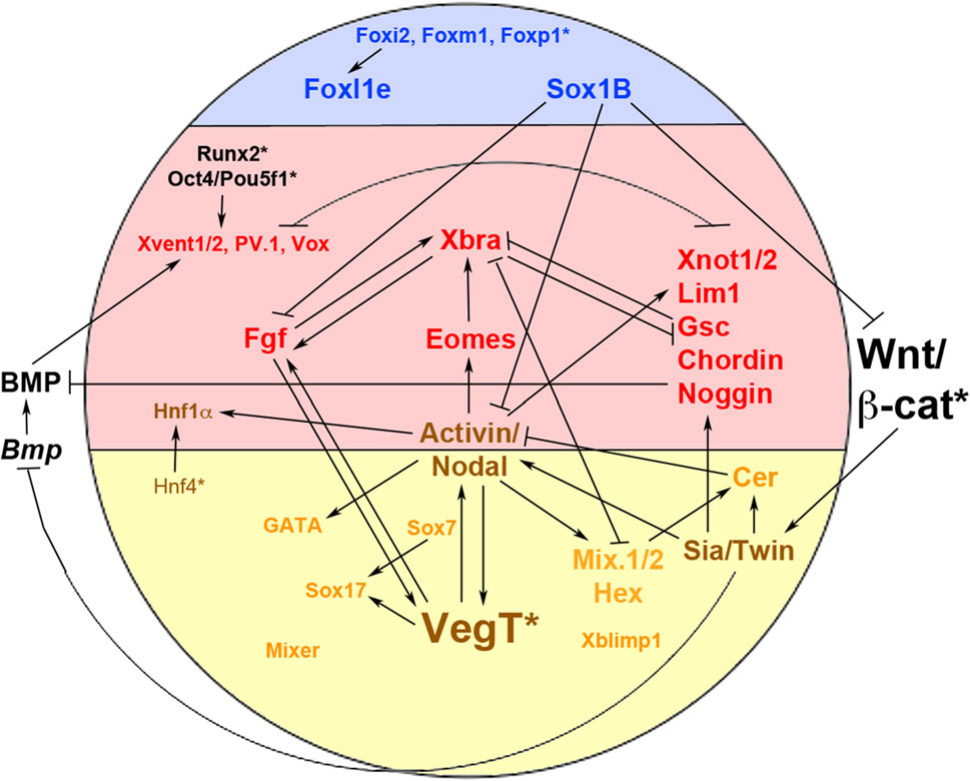
The gene regulatory network of mesendoderm formation. Schematic diagram of an amphibian blastula (dorsal to the *right*) showing a subset of the genetic interactions involved in germ layer formation. Genes expressed in the ectoderm are shown in *blue*, mesoderm in *red*, endoderm in *yellow*/*orange*; genes expressed in both endoderm and mesoderm are shown in *brown*; more generally expressed genes in *black*. Key maternal factors are indicated with an *asterisk*

After the onset of zygotic transcription there is a DV wave of *VegT* expression in the equatorial region of the blastula, and ectopic expression can be induced by eFGF (but not bFGF), Nodal/Activin signalling and by itself at this stage [21, 145, 146]. Thus, maternal VegT not only initiates a cascade of mesendoderm induction (Fig. 3), but also maintains and augments its own expression via positive feedback mechanisms. It has been suggested that maternal *VegT* mRNA is present in a vegetal-animal gradient with high doses of this factor inducing endoderm and lower doses inducing mesoderm [150].

VegT is required for the expression of FGFs and of the TGFβs nr1/2/4/5/6 and Derrière, and overexpression of each of these TGFβs in VegT-depleted embryos rescues different aspects of mesoderm and endoderm induction, indicating that VegT functions in a superordinate fashion with respect to these pathways [78, 107, 111, 112]. As mentioned previously, the induction of *nr5* and *nr6* occurs earlier than the onset of zygotic transcription of most other genes [24, 151], but *VegT* plays an ongoing role in mesodermal differentiation as it is required for the formation of the paraxial mesoderm which gives rise to the somites and muscle at later stages [152].

### Hepatocyte nuclear factors

Another group of transcription factors potentially involved in mesendoderm specification in *Xenopus* are the Hepatocyte nuclear factor (Hnf) family. Hnf4 is present as a maternal protein that is enriched in the vegetal half of the oocyte and has been shown to activate *Hnf1α* , a marker of definitive mesendodermal lineages (liver, gall bladder, gut, pronephros etc. [153]). Interestingly, the Hnf4 binding site in the *Hnf1α* promoter is in close proximity to an Activin-response element (ARE, binds Smad2/3), suggesting that Activin/Nodal signalling and maternal Hnf4 cooperate in activating mesendodermal gene expression [44]. Experiments using a dominant-negative form of Hnf1β suggest that Hnf1 activity is required for mesendoderm formation. This activity depends on Nodal/Activin, but not on FGF, signalling as (1) ectopic expression of Hnf1β alone does not result in mesoderm induction and (2) the dominant-negative construct blocks Vg1-mediated, but not eFGF-mediated, mesendoderm induction in animal cap explants [154].

### Transcriptional targets of the maternal β-catenin signal

Besides inducing *nr5* and *nr6* and repressing *bmp4* (see above), maternal β-catenin has several other target genes that encode transcription factors. The first gene proposed to be a marker of the ‘Nieuwkoop Centre’ was the homeobox gene *siamois* (*sia*), which is detected shortly after the onset of zygotic transcription in dorsovegetal cells of the blastula embryo [110, 155]. Sia, a potent inducer of ectopic embryonic axes [156], is induced by maternal Wnt/β-catenin signalling independently from mesoderm formation (Fig. 3) [157–160]. *Sia* functions redundantly with its close relative, *twin*, which is also induced directly by Wnt signalling and also induces secondary embryonic axes [161]. Sia and Twin proteins form both homodimers and heterodimers and their simultaneous (but not individual) knockdown results in disrupted organiser and axis formation [162].

One of the direct transcriptional targets of Sia/Twin is *cerberus*, encoding a multifunctional growth factor inhibitor related to Coco. *Cerberus* is expressed in the anterior endoderm of Spemann’s organiser and promotes head formation via inhibiting the posteriorising/ventralising Nodal, BMP and Wnt pathways during gastrulation [163–165]. As Sia and Twin also repress *bmp4* expression, their function in dorsalising the embryo is likely to be twofold: (1) they repress the transcription of ventralising *bmps* and (2) they induce dorsalising organiser factors such as *cerberus* and *chordin* [155].

The *bozozok*/*dharma*/*nieuwkoid* (*boz*) gene is the functional equivalent of *sia* in the zebrafish embryo, although their sequences are too divergent to be true orthologues [166]. As for *sia* in the frog, *boz* is induced by maternal Wnt/β-catenin activity on the dorsal side of the embryo, and it is required to repress the expression of *bmp2b* dorsally, in the prospective organiser region [167, 168]. The *boz* promoter has several high-affinity binding sites for Wnt transducers of the TCF/LEF family, emphasising that it is a likely direct target of the dorsalising maternal pathway [168]. Recently, it was demonstrated that the Boz protein is a target of the E3 ubiquitin ligase Lnx-l (Ligand of Numb protein-X-like). Depletion of Lnx-l results in increased Boz levels and strongly dorsalised embryos, indicating that the proteolytic turnover of Boz plays a central regulatory function in DV axis formation in fish [169].

### Transcription factors in mesoderm formation: *Brachyury*

The first gene found to be essential for mesoderm formation was *Brachyury* (*Short Tail, T*) in the mouse. *Brachyury* is a naturally occurring mutation that affects tail length and sacral vertebrae in heterozygous mice [170]. Homozygous animals die during embryogenesis due to severe defects in mesoderm formation and morphogenesis [171–173]. The cloning of the *T* locus was one of the first examples of positional gene cloning [174], and its gene product was found to be expressed in the primitive streak, the prospective mesoderm and the notochord of the early mouse embryo [175]. The *Xenopus* orthologue of *Brachyury, bra* (also known as *Xbra*), was identified shortly afterwards and was detected equatorially—as expected for a mesodermal determinant—where it is induced by Nodal/Activin signalling [176, 177]. Subsequently Brachyury’s DNA-binding activity was discovered [178], it was revealed that it functions as a tissue-specific transcription factor [179], and overexpression of *bra* in *Xenopus* resulted in widespread ectopic induction of mesoderm [180]. Conversely, injection of mRNA encoding a dominant-negative version of bra interfered with mesoderm formation in both frog and fish embryos, to some extent replicating the mutant phenotype in the mouse [181]. Everything pointed towards a key role for *Brachyury* in mesoderm formation.

In follow-up studies *bra* was shown to be induced by FGF signals [19, 182, 183] and, conversely, bra induced expression of *efgf* and required a functional FGF signalling pathway in order to induce mesoderm [184]. Thus *bra* and the mesodermal competence signal FGF activate each other in a positive feedback loop (Fig. 3). *Bra* is induced by low levels, but repressed by high levels of Activin, and it has been proposed that this dose-dependency of *bra* induction provides a read-out of the Nodal/Activin gradient that patterns the mesoderm [183, 185–187].

A screen for transcriptional targets of bra led to the identification of five genes: *bix1*, *wnt11*, *egr1*, *btg1* and *BIG3*/*1A11* [188]. *Bix1*, a paired box-homeodomain transcription factor, is a direct target of bra and induces ventral mesoderm and endoderm following mis-expression at low and high levels, respectively [189]. Wnt11 activates a β-catenin-independent, non-canonical Wnt pathway that orchestrates gastrulation movements [190]. *Egr1* encodes a zinc finger transcription factor expressed throughout the mesoderm in a DV wave. The induction of *egr1* by bra is likely to be indirectly mediated via the FGF-MAPK signalling pathway [191].

### Transcription factors in mesoderm formation: *Eomesodermin*

Yet another T-box gene called *Eomesodermin* (*Eomes*), whose expression precedes that of *bra*, was identified by Sir John Gurdon’s laboratory in 1996. *Eomes* is induced by TGFβs (but not eFGF) and its overexpression in animal cap tissue results in induction of a broad range of mesodermal markers, including *bra* [192]. This places *Eomes* upstream of *bra* in the mesoderm induction cascade. The *Eomes* promoter contains an ARE indicating that its expression is a direct result of TGFβ-mediated mesendoderm induction. It also contains a repressor element that blocks *Eomes* expression in the endoderm [192, 193].

Targeted disruption of the *Eomes* locus in the mouse revealed that this gene is also essential for mesoderm formation in mammals [194]. In zebrafish *Eomes* is expressed maternally underlining its importance in the earliest steps of embryo patterning; gain- and loss-of-function experiments suggested a slightly different role for Eomes in establishing the organiser (dorsal mesoderm) [195]. In addition Eomes is also required for endoderm formation in the fish embryo [196].

### Transcription factors specifying the dorsal mesoderm

There are a large number of transcription factors that are differentially expressed in DV subdivisions of the mesoderm. The dorsal-most mesoderm (Spemann’s organiser) expresses the homeobox genes *goosecoid* (*gsc*), *not1, not2, Xenopus iroquois 1* (*iro1*), the LIM/homeobox gene *lim1* and the winged-helix genes *pintallavis*/*foxA4a* and *Hnf3*β/*Foxa2* [197–203]. The homeobox genes *vent1, vent2*, *PV.1, vox,* the basic helix-loop-helix gene *myoD* and the zinc finger gene *Xpo* are expressed in a complementary fashion in the ventrolateral mesoderm [204–209]. The myogenic factor *myf5* is expressed in the paraxial mesoderm, overlapping with many of the ventrolaterally expressed genes, but excluding the dorsal-most region [210].

Gsc represses *bra*, and it has been suggested that this is how different Activin doses are translated into different DV gene expression domains in the mesoderm [183]. *Gsc*, *not1*/*2*, *lim1* and *pintallavis* are all induced by Activin, but only *not1*/*2*’s expression also depends on FGF signalling (but see [35, 198]). Consistently an ARE was identified in *lim1*’s first intron [211]. Loss-of-function experiments in frog, fish and mouse embryos have revealed that most of the dorsally expressed transcription factors are required at least to some extent for dorsal mesoderm formation [73, 212–216]. Importantly mice lacking *Lim1* function are headless [217] and those lacking Foxa2 fail to form an organiser and a notochord [218, 219] suggesting a key role for Lim1 in head development and a role for Foxa2 dorsal specification. However, mice with a targeted mutation in *Gsc* gastrulate normally and instead display craniofacial defects and malformations of the rib cage, suggesting that *Gsc* is not essential for organiser function—at least in mammals [220, 221]. Yet, depletion of *gsc* mRNA in frog embryos results in anterior defects, suggesting differential requirements for this factor in rodents and amphibians [216].

A recent study in *Xenopus* found *Foxa2* expression throughout the endoderm at late blastula stages, and overexpression experiments using different gain-of-function and antimorphic constructs suggested that endodermal *Foxa2* antagonises dorsal mesoderm and axis formation. The authors propose that this parallels a requirement for this gene in the extraembryonic endoderm in the mouse and that a conserved role of *Foxa2* is to limit ectopic mesoderm formation [222, 223].

### Transcription factors specifying the ventrolateral mesoderm

The first factor found to be expressed in the ventral mesoderm at the onset of gastrulation in *Xenopus* was the basic helix-loop-helix transcription factor MyoD [204]. In mouse MyoD was initially identified as a main regulator of muscle formation that commits cells to the myogenic lineage upon transfection [224]. *Xenopus myoD* is expressed throughout the ventral mesoderm, including non-myogenic mesoderm, at the onset of gastrulation, suggesting that transient expression of this factor is not sufficient to induce myogenesis and that it is a more general response to ventral mesoderm induction [204].

*Xenopus myf5* is a relative of *myoD* as it also encodes a basic helix-loop-helix transcription factor that is able to convert mammalian cells into muscle upon transfection [225]. Unlike *myoD, myf5* is expressed only in the paraxial mesoderm (presumptive somites) lateral of the organiser region [210].

The homeobox genes *vent1*/*2, PV.1* and *vox* are all expressed in the ventrolateral mesoderm; they are induced by ventralising BMP signalling; and they induce BMPs, antagonise organiser genes and ventralise embryos upon overexpression [32, 205–208]. Injections of dominant-negative forms of these factors into ventral blastomeres of *Xenopus* embryos frequently result in the induction of a secondary organiser and, subsequently, body axis duplication [226, 227].

The *vent* and *vox* genes are also found in zebrafish where they are called *vega2* and *vega1*, respectively. They exert the same function as in frogs: (1) induction of ventral fates and (2) antagonism of the organiser by establishing a cross-repressive loop with *boz* [228, 229]. Another homeobox gene, *ved*, and the zebrafish *even skipped*-like gene *eve1* also function in this group of ventralising factors [230, 231].

Zebrafish maternal and zygotic *spiel ohne grenzen* (*MZspg*) mutants display a profound expansion of dorsal at the expense of ventral tissues at the onset of gastrulation [232]. The mammalian orthologue of the *spg* gene is *Oct4*/*Pou5f1*, a known pluripotency or ‘stem cell’ factor, and *vox* is one of zebrafish Pou5f1’s direct transcriptional targets. Furthermore, Pou5f1 negatively controls *fgf8* expression [233]. These findings demonstrate that the acquisition of ventral identity also involves an active early (maternal) step.

### Transcription factors in endoderm formation: the Mix/Mixer/Milk family

Depletion of maternal VegT from *Xenopus* oocytes demonstrated that this factor is strictly required for both mesoderm and endoderm formation (see above), raising the question as to which factors mediate the specification of the endoderm.

The first transcription factor found to be expressed in the endoderm (although not exclusively, as it is also expressed in the mesoderm) of the *Xenopus* embryo was the homeodomain protein Mix.1 which is directly induced by TGFβ and FGF signalling in animal cap explants. In fact, this was the first study suggesting that the signals inducing mesoderm and endoderm may be similar [234]. Interestingly Mix.1 can heterodimerise with Sia and may antagonise Sia function in embryonic axis induction, although the functional relevance of this is somewhat unclear [235]. Mix.1 also suppresses *bra* suggesting that high levels of Mix.1 may promote endoderm at the expense of mesoderm induction [183].

Lemaire et al. reconciled these observations by demonstrating that Mix.1 and bra mutually repress each other and that Mix.1 synergises with Sia in inducing the endodermal marker genes *cerberus, endodermin* (*edd*) and *Xlhbox8* in animal caps [163, 236–238]. Importantly, blocking Mix.1 function using a dominant-negative construct resulted in a loss of endoderm differentiation [214, 238, 239]. Another factor cooperating with Mix.1 is the zinc finger transcription factor blimp1; these two factors synergise in blocking trunk mesoderm and promoting anterior endoderm formation [240]. Collectively these experiments provided support for the idea that Mix.1 is a key factor in specifying the endoderm.

Several *mix.1*-related genes with very similar characteristics were subsequently identified: *mix.2, bix2*/*milk, mixer*/*mix.3,bix1*/*mix.4, bix3* and *bix4* [241, 242]. Of those four genes, the expression of *mixer* is confined to the endoderm, suggesting a specific role in its induction [242], whereas *bix1* induces both endoderm and ventral mesoderm in overexpression experiments [189]. *Bix4* is the only maternally expressed gene of this group (in both mesoderm and endoderm). Its subsequent zygotic induction requires maternal VegT, and it rescues endoderm formation, but not mesoderm induction, in VegT-depleted embryos, indicating that it plays an essential role in endoderm formation downstream of VegT [243].

The zebrafish *bonnie and clyde* (*bon*) locus encodes a zebrafish Mix-related transcription factor, and *bon* mutants display a severe reduction of endodermal precursor cells [244]. Another Mix-like factor, Mezzo, is acting in parallel with Bon in fish, highlighting the importance of this gene family in endoderm formation [245]. The promoter of *mix.2* has a well-characterised ARE, confirming that *mix* genes are a direct response to mesendoderm-inducing Nodal/Activin signalling [246].

### Transcription factors in endoderm formation: the Sox family

The high mobility group (HMG) transcription factors Sox17α and Sox17β are two of the more specific markers of endoderm in *Xenopus* embryos. Their overexpression in animal cap explants induces markers of the definitive endoderm and this induction can be blocked by a dominant-negative Sox17 protein. The dominant-negative also inhibits the induction of endoderm by activin, indicating that Sox17α/β function downstream of this inducer, and it blocks endoderm formation in whole embryos [45]. Experiments using specific VegT and Nodal inhibitors suggested that Sox17α is induced by both factors, but in successive time windows: the initial induction of Sox17α depends on VegT, but not on Nodal signalling, whereas the maintenance of Sox17α expression during gastrulation requires both VegT and Nodal [247].

Interestingly Sox17α/β antagonise the dorsalising maternal Wnt/β-catenin signal upstream of *sia* induction. This inhibition is likely due to a physical association of the Sox17 proteins with β-catenin [248]. It has been suggested that, by interacting with β-catenin, the SoxF subfamily of transcription factors (that includes Sox17) establishes at least some aspects of endodermal gene expression [249].

Another *Sox* gene of the *SoxF* subfamily that was found to be maternally expressed and localised to the vegetal hemisphere in *Xenopus, Sox7*, functions immediately downstream of VegT where it induces transcription of *nr1*/*2*/*4*/*5*/*6, mixer* and *Sox17*β. The *nr5* promoter contains a binding site that can be occupied alternatively by Sox3 or Sox7, but not by Sox17 [250]. Sox3, a member of the SoxB1 subfamily, is expressed in the animal hemisphere of the embryo where it antagonises *nr5* induction [251], suggesting that the animal–vegetal axis of the embryo is specified by the complementary expression of *SoxB1* and *SoxF* genes that repress and promote mesendoderm formation, respectively [252].

### Transcription factors in endoderm formation: GATA and Hex

Zinc finger transcription factors of the GATA family are also involved in endoderm formation in the frog. GATA4, GATA5 and GATA6 are all induced by Nodal/Activin signalling and they induce various endodermal marker genes in overexpression assay [253]. Consistently zebrafish GATA5, encoded by the *faust* locus, is required for endoderm formation in fish [254].

The homeobox gene *hex* was discovered as a marker of the anterior endoderm, the tissue that also expresses the multifunctional BMP/Nodal and Wnt inhibitor Cerberus. Intriguingly, *hex*-expressing cells originate in the blastocoel floor and move towards the anterior side at the onset of gastrulation. Ectopic expression of *hex* on the ventral side of a frog embryo resulted in axis duplication although the organiser markers *gsc* and *chordin* are downregulated by overexpression of *hex* on the dorsal side [255]. However, *hex* does induce *cerberus*, and blocking *hex* function using a dominant-negative construct inverts this scenario by repressing *cerberus* and upregulating *gsc* and *chordin*. Thus, *hex* promotes anterior endoderm at the expense of organiser formation [256]. More recently, it was shown that Hex boosts the early dorsalising Wnt signal by inhibiting the expression of *tle4*, encoding a Groucho-type co-repressor that blocks Wnt target genes. Consequently, *sia* and *nr3* are upregulated in areas of *hex* expression. Furthermore, Hex upregulates *nr1* and *nr2* expression directly [257].

How do mesoderm and endoderm become properly segregated after their initial induction? Many of the germ layer-specific transcription factors cross-repress each other, thereby stabilising this binary cell fate choice [238]. In some cases, these factors may play an even more direct role in segregating different cell populations: a recent study has revealed that the endoderm-specific factor Sox17 is required for the formation of the basement membrane that separates the gut endoderm from the mesoderm [258].

### Transcription factors in the ectoderm

Traditionally ectoderm was regarded as somewhat of a blank canvas—a ‘default state’ that can be turned into mesoderm or endoderm via the addition of the right inducers. We have above discussed the roles of ectodermally expressed growth factor antagonists (Coco, norrin and Ectodermin) in protecting ectodermal cell fate from excessive signalling by such inducers. However, more recently factors that actively specify ectodermal cell fates have been identified.

Several *Forkhead box* genes are maternally expressed in *Xenopus* and their transcripts are localised to animal blastomeres: *foxi2*, *foxm1* and *foxp1* [259]. *Foxl1e* (also known as *Xema, Xenopus ectodermally expressed mesendoderm antagonist*), a zygotically expressed *foxi* gene encoding an inhibitor of mesendoderm induction [260], is a direct target of Foxi2 [261]. Overexpression of *foxl1e* results in suppression of mesendodermal identity whereas *foxl1e* knockdown causes the opposite effect with various mesendodermal markers encroaching upon the animal hemisphere [260]. In addition *foxl1e* is required in a cell-autonomous fashion for the maintenance of ectodermal fate, as Foxl1e-depleted ectoderm cells intermingle with other germ layers and subsequently differentiate according to their new positions [262].

As mentioned above, the *SoxB1* gene *Sox3* is expressed in the animal hemisphere of the frog embryo where it antagonises mesendoderm formation by repressing *nr5* [251]. Like *Sox17*, *Sox3* also antagonises the dorsalising early Wnt signal by directly interacting with β-catenin [248]. Similarly, the *SoxB1* genes *Sox3* and *Sox19a*/*b* antagonise the cascade that leads to organiser formation downstream of maternal Wnt signalling in zebrafish embryos [263]. Moreover, *SoxB1* genes restrict dorsal mesoderm formation by repressing the expression of *fgf3* and *fgf8* [264]. Thus, the SoxB1 family protects ectodermal fate (1) by antagonising mesendoderm induction by SoxFs and (2) by limiting dorsal mesoderm formation [252].

Taken together, complex networks of transcription factors regulate the formation of all germ layers. These networks contain negative and positive feedback loops that serve to stabilise cell fate decisions. It is striking that certain families of transcription factors appear to be selectively overrepresented in specific aspects of germ layer formation—the Foxi and SoxB1 families in the ectoderm, the GATA and SoxF families in the endoderm and the Mix homeodomain and T-box families in the mesendoderm (Fig. 3).

## Competence

The idea that cell specification during embryogenesis is mediated by inductive events between tissues goes back to the very beginnings of experimental embryology. However, already at that time it was noted that a tissue receiving an inductive signal has to be competent to respond to the signal in a specific manner [265]. This concept of ‘embryonic competence’ persists until today and has some relevance for the induction of the germ layers.

### Competence for mesendoderm induction

In 1985, Dale et al. noted that the competence for mesoderm induction in animal cap explants depends on the developmental stage of the donor embryo: animal caps dissected at blastula stage can be converted into mesoderm whereas animal caps dissected at early gastrula stage have lost this competence [266]. This loss of mesodermal competence occurs in dissociated cells and even in the presence of the protein synthesis inhibitor cycloheximide, indicating that it is a cell-autonomous process that does not require cell communication, proliferation or new protein synthesis [267]. Similarly a loss of competence for the dorsalising signal was observed during gastrulation [268].

A compellingly simple model for the loss of responsiveness to Nodal/Activin signalling was proposed by the Gurdon laboratory: translocation of the Nodal/Activin transducer Smad2 is a prerequisite for the transduction of a signal, and increased phosphorylation of Smad2 at a site distinct from its activation domain after the onset of zygotic transcription prevents Smad2 from being shuttled into the nucleus. Thus, the loss of competence for mesendoderm inducers of the Nodal/Activin family is due to nuclear exclusion of Smad2 [269].

A landmark study in 1997 demonstrated that the onset of zygotic transcription is accompanied by an exchange of linker histones—proteins ensuring that chromosomal DNA stays tightly wrapped around the core histones—from an oocyte-specific form, histone B4/H1M, to histone H1, and that this exchange is causative for the loss of mesodermal competence [270]. This was one of the first studies implicating an epigenetic mechanism in a developmental cell fate decision. More recently, a finely tuned balance of different linker histone variants was found to endow blastomeres with mesodermal competence [271].

Another histone that is differentially expressed during early embryogenesis is the core histone H3.3. Depletion of H3.3, or of the H3.3 chaperone HIRA, results in mesodermal defects; however, these are likely to emerge after mesoderm induction has occurred, as the earliest marker whose expression is affected is *bra* whereas earlier markers such as *eomes* remain unchanged [272].

Although ectodermal cells have lost the competence to form mesoderm in response to Activin/Nodal and FGF at late gastrula stages, experiments with an inducible bra constructs demonstrated that ectopic expression of this factor still converts them into mesoderm, consistent with the later function of bra in the mesoderm induction cascade [273]. A series of grafting experiments using more differentiated mesodermal tissues also indicated that germ layer identities are not fixed until the end of gastrulation [274]. Experimental expression of an activated form of Smad2 in the neuroectoderm resulted in suppression of *SoxB1* genes and concomittant induction of *chordin* and *myoD*, lending further support to the plasticity of germ layer identities during gastrulation [275]. Thus, the loss of mesodermal competence can be overridden under specific circumstances.

An interesting recent study has revealed that blastula stage animal pole cells express a range of molecular markers that later on are also expressed in the neural crest, a multipotent cell population that migrates into different locations of the developing embryo and gives rise to multiple cell types. Activin treatment induces endoderm from neural crest cells, suggesting that the overlapping set of factors that is expressed in both animal cap and neural crest cells may function as a conserved set of multipotency factors that endow these cells with the competence for endoderm induction [276].

### Dorsoventral competence

Shortly after the discovery of Activin it was noted that animal blastomeres exhibit a pre-pattern that determines the response to this inducer: dorsal and ventral animal blastomeres treated with the same dose of Activin develop predominantly into trunk/tail structures and ventral mesoderm, respectively [104, 277]. Dorsal competence was later shown to be induced in ventral animal blastomeres by treatment with the pharmacological Wnt activator lithium [278], and it is now clear that activin treatment of animal blastomeres simply reveals the DV pattern that is induced by the dorsalising maternal Wnt/β-catenin signal discussed above [279]. Thus, spatial differences in competence may be caused by a pre-pattern that has been established previously, or by partially overlapping signals. Along similar lines, FGFs are now regarded as competence factors that allow mesoderm induction in response to mesendoderm inducers of the Nodal/Activin family, rather than mesoderm inducers themselves (see above) [33–35, 37, 280].

## Germ layer formation in amniotes

Amniotes (reptiles, birds and mammals) differ from anamniotes (fish and amphibians) in that their eggs develop either in ovo or in utero. This puts certain constraints on embryonic development, with consequences for the way the germ layers are formed in these animals. Because the embryos of oviparous amniotes tend to develop over a longer time frame than those of anamniotes, and because their eggs are laid out for terrestrial development, they need a larger supply of yolk and liquid. The disproportionally large yolk found in birds and reptiles (and in monotreme mammals) imposes morphological constraints on the shape of their embryos and, consequently, on the geometry of germ layer formation [281].

### Germ layer formation in birds and reptiles

**Fig. 4:**
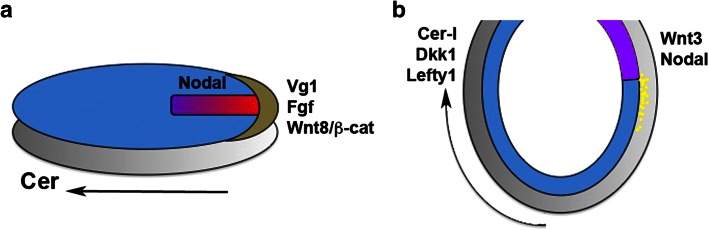
Germ layer specification and related gene expression in early gastrula chick (**a**) and mouse (**b**) embryos. Anterior points to the *left*, dorsal is up. The germ layers are *colour*-coded as in Fig. 1. Extraembryonic tissues (hypoblast in chick, visceral endoderm in mouse) are shown in *grey*, Koller’s sickle in *brown*. Note extension of the primitive streak from the posterior end of the embryo (*red*/*purple*) and simultaneous anterior displacement of the anterior hypoblast/AVE (*dark grey*). Endoderm (*yellow dots* in **b**) and mesoderm intercalate between the epiblast and the extraembryonic layers during gastrulation

At blastoderm stages, bird and reptile embryos consist of two layers: the superficial epiblast that gives rise to the embryo proper, and the hypoblast beneath it that does not contribute to the embryo itself. The beginning of gastrulation in birds is marked by a thickening of the epiblast on the presumptive posterior side (‘Koller’s sickle’), from which the macroscopically visible ‘primitive streak’ starts to extend anteriorly, towards the middle of the epiblast. During gastrulation, epiblast cells ingress through the primitive streak and insert themselves into the lower layers, thereby forming the endoderm and mesoderm of the avian embryo (Fig. 4a). Thus, the primitive streak is the equivalent of the blastopore of anamniote embryos.

There is evidence that the endoderm and the different derivatives of the mesoderm are specified by the time of their ingression through the streak: endoderm seems to originate from cells that ingress through the anterior part of the elongating streak [282], whereas mesoderm ingresses shortly thereafter with more dorsal mesoderm coming from the anterior, and more ventral mesoderm coming from the posterior streak [282, 283].

### TGFβs, FGFs and Wnts in birds

The role of TGFβs as the principal inducers of mesoderm is conserved in birds. Activin is expressed by the hypoblast at the time of mesoderm induction and exogenous Activin induces all types of mesodermal structures in isolated epiblast tissue and in chick embryos [47, 284, 285]. Exogenous Activin also induces *Brachyury*, which is normally expressed in the primitive streak and in early migrating mesoderm cells [179], and *Gnot1*, the chick orthologue of *not1* [286].

Chick Vg1 expression is more localised than that of Activin and is restricted to Koller’s sickle. Application of Vg1 to ectopic locations in the marginal zone of the epiblast resulted in induction of complete secondary streaks, suggesting that Koller’s sickle could be the functional equivalent of the ‘Nieuwkoop Centre’ [60, 61].

Chick Nodal is expressed in the posterior epiblast, immediately adjacent to the domain of Vg1 expression, and later on in the primitive streak [287–289]. Importantly, Nodal expression is induced in response to ectopic Vg1, suggesting a relay of signals whereby Vg1 in Koller’s sickle induces Nodal in the epiblast [290]. Like Vg1, ectopic Nodal can induce a primitive streak, but only after the hypoblast has been removed. The hypoblast expresses the chick orthologue of Cerberus (a BMP/Wnt/Nodal inhibitor), and the Nodal-specific short form Cer-S mimics the streak-inhibiting function of the hypoblast. Thus, the site of primitive streak formation is confined to the posterior side of the embryo by the anti-Nodal activity of the anteriorly moving hypoblast (Fig. 4a) [291].

FGFs are expressed before the onset of gastrulation [289] and functional FGF signalling is required for mesoderm formation in chick [47]. However, FGFs also play an important role in neural induction in the chick embryo [292, 293]. The switch between mesoderm and neural induction by FGF is mediated by the zinc finger protein Churchill which is induced by FGF and activates Smad interacting protein 1 (SIP1) which subsequently blocks mesoderm induction [294].

Increased nuclear localisation of β-catenin marks the dorsal side of frog and fish embryos at blastula stages [89]. Nuclear localisation of β-catenin is also observed before the onset of gastrulation in chick: initially it is found radially in the marginal zone of the epiblast, but with the appearance of Koller’s sickle it is also seen in endoblast cells that spread out beneath the epiblast from posterior to anterior [295]. Thus, despite their morphological differences, β-catenin activation appears to mark the site of organiser formation in both anamniotes and amniotes. Remarkably Cerberus (expressed in the hypoblast), has the ability to inhibit Wnt as well as Nodal signalling; thus, it is conceivable that the anterior displacement of the hypoblast by the endoblast positions the primitive streak via a dual inhibition mechanism involving both Wnt and Nodal signalling.

In anamniotes the mesoderm forms in a circumblastoporal ring that surrounds the entirety of the embryo whereas it only forms on the posterior side in amniotes. A recent study suggested that it is the posterior restriction of mesoderm-inducing FGF and Wnt signals that limit mesoderm induction to the streak in amniotes. Indeed, exogenous application of FGF protein to amniote embryos results in mesoderm induction throughout the entire marginal zone around the epiblast and a concomitant reduction of the streak [24], substantiating the previously suggested relationship between the anamniote blastopore lip and the amniote primitive streak [281].

### Germ layer formation in the mouse

The pre-gastrula stage mouse embryo is cup-shaped and consists of an extraembryonic outer layer, the visceral endoderm, and an inner layer, the epiblast that gives rise to the embryo proper. Gastrulation begins on one side of the cup, marking the future posterior side of the embryo, and involves the formation of a primitive streak with the organiser (node) at its tip [296]. Prior to gastrulation, visceral endoderm that is located at the distal tip of the cup moves to the anterior side, similar to the anterior movement of the hypoblast in the chick. This anterior visceral endoderm (AVE) expresses the Nodal antagonists Cerberus-like and Lefty1 and the Wnt inhibitor Dickkopf1, confining Nodal and Wnt signalling to the posterior side of the cup (Fig. 4b) [297, 298]. Thus, despite different morphologies, chick and mouse embryos are rather similar with the chick hypoblast being the equivalent of the murine visceral endoderm [291].

Nodal is strictly required for mesendoderm formation in the mouse [65, 66]; however, the expression of Nodal in the early mouse embryo is surprisingly dynamic [299], suggesting that other factors restrict mesendoderm induction in the mouse. Unlike in other vertebrate models, BMP4 is also required for mesoderm formation in the mouse [300].

Similar to the other vertebrate models, early Wnt signalling appears to play an important role in breaking embryonic symmetry in the mouse [298]. Both genetic loss of the Wnt inhibitor Axin and transgenic overexpression of chick Wnt8c result in the formation of multiple primitive streaks—similar to the effect of ectopic Wnt expression in frog and fish [301]. *Wnt3* is expressed in the right location and early enough to provide this axis-inducing signal, and its disruption results in a complete absence of mesoderm, primitive streak and node. Accordingly, Nodal is not expressed in *Wnt3* knockout mice [302]. This is in contrast to anamniote embryos where the dorsalising Wnt/β-catenin signal can largely be uncoupled from the mesendoderm-inducing Nodal signal (see above).

Taken together, despite differing embryonic geometries, most of the key factors that mediate germ layer specification are conserved between anamniotes and amniotes.

## Targeted programming of stem cells

The targeted differentiation of embryonic stem cells (ESCs) into virtually any tissue type holds great therapeutic promise for regenerative medicine. A thorough understanding as to how a tissue is generated in the developing organism can help us to recapitulate the underlying developmental programme in vitro. Since germ layer specification is the first step in the differentiation of all embryonic tissues, our knowledge of the GRNs that specify the endoderm, mesoderm and ectoderm is fundamental to devise sound stem cell differentiation protocols.

The major signalling pathways involved in mesendoderm specification in vertebrate embryos also result in the induction of comparable cell fates in ESCs: addition of either BMP4 or Wnt protein to ESC cultures results in the formation of primitive streak-like mesoderm [303–305] whereas Nodal induces mesoderm at lower and endoderm at higher doses [306]. Wnt signalling is not only sufficient, but also necessary for ESC mesoderm induction [307]. In contrast, BMP4 is not required suggesting that its ability to induce mesoderm is mediated indirectly via the induction of other mesoderm inducers (Nodal and Wnts) (reviewed in [308]).

Using geometrically defined cultures of human stem cells, the Brivanlou lab recently showed that, if these cells are grown in the shape of a disc, they respond to treatment with BMP4 by forming concentric circles of ectoderm, mesoderm and extraembryonic trophectoderm, essentially recapitulating the spatial arrangement found in mammalian embryos. In these experiments they also found that the effect of BMP4 is mediated via the induction of Activin/Nodal signalling that is induced in an increasing inside-out gradient [309]. Thus, by combining developmental signals with geometric constraints, the earliest steps of embryonic development can be fairly faithfully recapitulated in a dish.

## Concluding remarks

Pioneering studies in amphibian embryos paved the way for current work aiming to assemble the GRN of germ layer specification in different species. Diffusible signals (such as the Nodals and FGFs) and a few maternal transcription factors are required for the initial specification of the germ layers. The combination of these induces germ layer-specific sets of transcription factors that regulate the subsequent acquisition and segregation of cell fates. Many of these factors cross-repress each other, which stabilises alternative cell fate decisions.

Although the formation of the germ layers is the earliest step in cell fate specification, the underlying GRN that mediates this decision is bafflingly complex, involving multiple secreted signalling factors and cascades of transcription factors many of which cross-regulate each other. The knowledge of this network is now being employed to drive stem cells along desired routes of specification.
